# Clinical Significance of the CCR5delta32 Allele in Hepatitis C

**DOI:** 10.1371/journal.pone.0106424

**Published:** 2014-09-05

**Authors:** Isabelle Morard, Sophie Clément, Alexandra Calmy, Alessandra Mangia, Andrea Cerny, Andrea De Gottardi, Meri Gorgievski, Markus Heim, Raffaele Malinverni, Darius Moradpour, Beat Müllhaupt, David Semela, Stéphanie Pascarella, Pierre-Yves Bochud, Franco Negro, on behalf of the Swiss Hepatitis C Cohort Study Group

**Affiliations:** 1 Division of Gastroenterology and Hepatology, University Hospitals, Geneva, Switzerland; 2 Division of Clinical Pathology, University Hospitals, Geneva, Switzerland; 3 Division of Infectious Diseases, University Hospitals, Geneva, Switzerland; 4 Liver Unit, IRCCS "Casa Sollievo della Sofferenza," San Giovanni Rotondo, Italy; 5 Service of Hepatology, Clinica Moncucco, Lugano, Switzerland; 6 Division of Hepatology, University Hospital of Bern, Bern, Switzerland; 7 Division of Infectious Diseases, University Hospital of Bern, Bern, Switzerland; 8 Division of Gastroenterology and Hepatology, University Hospitals of Basel, Basel, Switzerland; 9 Division of Infectious Diseases, Pourtalès Hospital, Neuchâtel, Switzerland; 10 Division of Gastroenterology and Hepatology, University Hospital and University of Lausanne, Lausanne, Switzerland; 11 Division of Gastroenterology and Hepatology, University Hospitals of Zurich, Zurich, Switzerland; 12 Division of Gastroenterology, Canton Hospital, St. Gall, Switzerland; 13 Department of Pathology and Immunology, University of Geneva, Geneva, Switzerland; 14 Division of Infectious Diseases, University Hospital and University of Lausanne, Lausanne, Switzerland; University of Navarra School of Medicine and Center for Applied Medical Research (CIMA), Spain

## Abstract

**Background:**

The CCR5 receptor, expressed on Th1 cells, may influence clinical outcomes of HCV infection. We explored a possible link between a *CCR5* 32-base deletion (CCR5delta32), resulting in the expression of a non-functioning receptor, and clinical outcomes of HCV infection.

**Methods:**

*CCR5* and HCV-related phenotypes were analysed in 1,290 chronically infected patients and 160 patients with spontaneous clearance.

**Results:**

Carriage of the CCR5delta32 allele was observed in 11% of spontaneous clearers compared to 17% of chronically infected patients (OR = 0.59, 95% CI interval 0.35–0.99, P = 0.047). Carriage of this allele also tended to be observed more frequently among patients with liver inflammation (19%) compared to those without inflammation (15%, OR = 1.38, 95% CI interval 0.99–1.95, P = 0.06). The CCR5delta32 was not associated with sustained virological response (P = 0.6), fibrosis stage (P = 0.8), or fibrosis progression rate (P = 0.4).

**Conclusions:**

The CCR5delta32 allele appears to be associated with a decreased rate of spontaneous HCV eradication, but not with hepatitis progression or response to antiviral therapy.

## Introduction

Hepatitis C virus (HCV) infects about 2.8% of the global population [1] and is a major cause of chronic liver disease and hepatic mortality worldwide [2], [3]. Although the treatment of HCV genotype 1, the most prevalent in western countries, still relies on the use of pegylated interferon-alpha (IFN-alpha) and ribavirin, with the addition of other direct-acting antivirals [2], [3], IFN-alpha-free regimens are currently being registered in several countries and for all viral genotypes [4]. The advent of these well-tolerated, highly efficacious drugs will revolutionize HCV screening strategies and the consequent assessment of treatment needs.

The factors influencing liver fibrosis progression and response to antivirals are complex and involve host, viral and environmental factors. Multiple host genetic polymorphisms affecting HCV-related liver inflammation, fibrosis progression and response to therapy have been reported encompassing a broad variety of genes, including *IFNL3*, associated with spontaneous and treatment-induced HCV clearance [5]–[9], as well as several genes recently associated with accelerated fibrosis progression rate [10], [11]. The clinical implications of these findings are considerable, since the future management of hepatitis C – especially the prioritization in costly treatment allocation – may increasingly depend on patient profiling, including host genetic polymorphisms.

The chemokine receptor CCR5 was identified as one of the major coreceptors for the human immunodeficiency virus-1 (HIV-1). A 32-bp deletion in exon 4 of *CCR5* (CCR5delta32) results in a non-functioning receptor that is trapped in the endoplasmic reticulum and therefore not expressed at the cell surface. This deletion occurs –at the homozygous state– in 0.7–1.6% of the Caucasian population: homozygous carriers are resistant to M-tropic strains of HIV-1 and a number of new anti-HIV drugs, called CCR5 receptor antagonists, have been designed to interfere with the interaction between CCR5 and HIV. CCR5 is expressed on many cell types, including Th1 cells [12] and hepatic stellate cells (HSCs) [13], [14], suggesting that this receptor may be important in viral clearance, response to therapy and fibrogenesis in chronic hepatitis C. Th1 response and increased CD8 T cell response via IFN-gamma production was associated with viral clearance and spontaneous recovery from acute HCV infection [15]. In contrast, the development of persistent chronic HCV infection has been correlated with an impaired Th1 response [15]–[17]. According to the hypothesis that CCR5 promotes the recruitment of Th1-expressing cells into the liver to mediate the clearance of HCV-infected hepatocytes, reduced expression of CCR5 should be associated with viral persistence. In case of chronic HCV infection we expect that the *CCR5* deletion should decrease liver inflammation and fibrosis. CCR5 is also recognized as an important mediator of pro-fibrogenic signalling in HSCs. Bruno *et al* [14] have shown that the HIV envelope glycoprotein gp120 directly acts on HSCs through the use of the CCR5 receptor, and one large study found a significant association between CCR5delta32 and reduced fibrosis [18]. However, the observational studies about CCR5 expression, spontaneous clearance, inflammatory activity during chronic HCV infection and the viral response to pegylated IFN-alpha and RBV treatment have provided controversial and conflicting results.

The aim of our study was to clarify the role of the CCR5delta32 allele in HCV-related outcomes, by performing comprehensive genotyping of *CCR5* in a large and diverse population of anti-HCV-positive persons and by correlating findings with the degree of liver inflammation, fibrosis stage, fibrosis progression rate, steatosis, HCV spontaneous clearance and response to IFN-alpha-based therapy. We also hypothesized that the results of this study may open new perspectives in the treatment of HCV-HIV coinfected patients with CCR5 antagonists such as maraviroc, a currently available anti-HIV drug.

## Materials and Methods

### Study patients

Patients were included from the Swiss Hepatitis C Cohort Study (SCCS), a multicenter study enrolling anti-HCV-positive persons at eight major Swiss hospitals since 2000 [19], [20] and from an Italian cohort (IC) contributed by the Liver Unit at the “Casa Sollievo della Sofferenza”, San Giovanni Rotondo, Italy. The study was reviewed and approved by the ethics committee of the Department of Medicine, Geneva University Hospitals, Geneva, Switzerland (protocol 2000-28) and the ethics comittee of IRCSS Casa Sollievo della Sofferenza, San Giovanni Rotondo. Only patients with available DNA and written informed consent for genetic studies were enrolled. Among 3,775 adult anti-HCV positive patients included in the SCCS up to March 2013, 1,332 had genomic DNA available which could be isolated and analyzed by PCR to identify CCR5delta32. An additional 118 adult anti-HCV positive patients were contributed from the IC, totalling 1,450 patients for the present study. We included both persons with spontaneous HCV clearance (defined as presence of anti-HCV but undetectable HCV RNA without history of previous antiviral treatment) and patients with chronic infection with HCV genotypes 1, 2, 3, or 4, treated or not with pegylated IFN-alpha and ribavirin. Demographic and clinical features (including age, gender, body mass index (BMI), risk factors for HCV acquisition, HCV genotypes, *IFNL3* rs12979860 polymorphisms, alcohol consumption, type 2 diabetes, markers of infection with HIV and hepatitis B virus, HCV RNA, transaminase levels, liver histology, and data on HCV treatment) were extracted from clinical databases. A sustained virological response (SVR) to anti-HCV therapy was defined as an undetectable HCV RNA in serum 24 weeks after treatment termination. Liver specimens were formalin-fixed and paraffin-embedded for histological evaluation and analyzed by experienced pathologists. Necroinflammatory activity and fibrosis were scored according to the Metavir scoring system [21]. *Significant* activity and fibrosis were defined as Metavir scores ≥A2 and ≥F2, respectively. *Severe* activity and fibrosis corresponded to Metavir scores of ≥A3 and ≥F3. Fibrosis progression rate was calculated in 921 patients with an estimated date of infection, as previously described [20]. Steatosis was defined as present (≥5% of hepatocytes) or absent.

### CCR5 genotyping

The presence of the CCR5delta32 deletion was assessed by PCR on DNA extracted from peripheral blood mononuclear cells drawn from all patients and processed by standard protocols. The following primers were used to amplify 274-bp (wild type) and 242-bp (delta32 deletion) fragments of *CCR5*, respectively: sense, 5′- TTTACCAGATCTCAAAAAGAAG -3′ and antisense, 5′- GGAGAAGGACAATGTTGTAGG -3′. The following conditions for the PCR reactions were used: 5 min at 95°C, 1 min at 95°C, 1 min at 57°C, 1 min at 72°C (30 cycles), and finally 5 min at 72°C. PCR products were analyzed on a 3% agarose gel.

### Statistical analyses

Statistical analyses were performed in Stata 12Software (StataCorp LP, College Station, Texas, USA). The association between CCR5delta32 and the different phenotypes (spontaneous clearance [both cohorts] and clinical and histological outcomes [SCCS dataset only] including hepatic inflammation, fibrosis, fibrosis progression rate and response to therapy) were assessed in univariate and multivariate regression models. Due to the relative rare allele frequency of CCR5delta32, the associations were assumed for an dominant mode of inheritance (patients heterozygous and homozygous for the rare alleles were compared to the other). Fibrosis progression rate was dichotomized using the median value, i.e. 0.08 fibrosis Metavir unit per year. Multivariate stepwise regression models (P<0.2) were used to determine the independent risk factors from the predicted variables.

## Results

### Patient population

The 1,450 patients' features are summarized in Table 1. Most patients were males (62%), all were Caucasians, and 160 (11%) were spontaneous clearers. The minor allele frequencies of CCR5delta32 and *IFNL3 rs12979860* were 8.6% and 36.8%, respectively. The frequency of CCR5delta32 was similar in patients with or without HIV coinfection (Fischer test, P = 0.87).

**Table 1:** Patient characteristics.

	Chronic infection	Spontaneous clearance
	N = 1290	N = 160
		
Male gender^1^	0.62	0.54
Caucasian ethnicity	1.0	1.0
Median age at infection (IQR)^2^	20 (9)	38 (19)
HCV reported risks		
Drug use	0.46	0.24
Invasive procedures/needle stick	0.24	0.22
Transfusion	0.18	0.29
Other/Unknown	0.13	0.24
HCV genotype^3^		
1	0.49	NA
2	0.09	NA
3	0.28	NA
4	0.10	NA
Alcohol consumption^4^		
>40 g/d for ≥5 years	0.21	NA
Diabetes	0.06	NA
Median BMI (kg/m^2^, IQR)^5^	24 (5)	NA
HIV+^6^	0.08	NA
Response to pegylated IFN-alpha and ribavirin therapy^7^	0.60	NA
Liver histological features		
Histological activity^8^		
A0	0.42	NA
A1-A3	0.58	NA
A0-A1	0.83	NA
A2-A3	0.17	NA
Fibrosis stage (Metavir)^9^		
F0-F1	0.44	NA
F2-F4	0.56	NA
Steatosis^10^	0.63	NA

Numbers are the proportion of patients with the indicated feature.

1 Gender was missing in 1 patient with spontaneous clearance.

2 Age at estimated date of infection for patients with chronic infection, at cohort entry for those with spontaneous clearance (missing in 2 patients).

3 HCV genotype was missing in most patients with spontaneous clearance and in 46 chronically infected patients.

4 Alcohol consumption data before treatment was missing in 18 patients.

5 BMI treatment was missing in 215 patients.

6 HIV serology was missing in 214 patients.

7 Response treatment was assessable in 693 patients.

8 Histological activity before treatment was missing in 279 patients.

9 Fibrosis stage before treatment was missing in 273 patients.

10 Steatosis data before treatment were missing in 155 patients.

The most frequently reported risk factor among chronically infected patients was the use of intravenous drugs (46%). Alcohol consumption >40 g/day in the 5 past years was reported in 21% of chronically infected patients, HIV coinfection in 8% and diabetes in 6%. Of note, none of the HIV-infected patients were carriers of the CCR5delta32 allele at the homozygote state.

Liver fibrosis was assessed at histology in 1,017 patients before any anti-HCV treatment susceptible to modify Metavir scores: 921 had an estimated infection date, making them assessable for the fibrosis progression rate estimation. Significant (≥A2) and severe (≥A3) inflammation were present in 58 and 17% of the patients, respectively, while significant (≥F2) and severe fibrosis (≥F3) were present in 56 and 27% of the patients, respectively, 16% of them having cirrhosis (F4). As expected, the median fibrosis progression rate was not normally distributed, with a median of 0.08 (interquartile range = 0.093), and a mean of 0.108 (range 0-1) fibrosis units per year. Six hundred ninety three patients were assessable for response to treatment with pegylated IFN-alpha and ribavirin, and 60% of them reached SVR.

### Chronic vs. spontaneously cleared HCV infection

Carriage of CCR5delta32 was observed in 11% of spontaneous clearers compared to 17% of chronically infected patients (OR = 0.59, 95% CI interval 0.35–0.99, P = 0.047). The association between CCR5 deletion and HCV spontaneous clearance was similar albeit less significant when patients with positive HIV serology were removed (P = 0.09) (Table 2). The association was slightly less significant in a multivariate model (OR = 0.60, 95% CI 0.35–0.99, P = 0.07), after adjustment for relevant covariates, including *IFNL3 rs12979860* (OR = 0.35, 95% CI 0.24–0.49, P = 2.8E-9), male sex (OR = 0.76, 95% CI 0.53–1.07, P = 0.12), and infection by invasive procedure or needle stick (OR = 1.61, 95% CI 0.98–2.63, P<0.001), blood transfusion (OR = 2.91, 95% CI 1.81–4.66, P = 0.06), or through another risk (OR = 3.55, 95% CI 2.17–5.81, P<0.001), compared to drug use (Table 3). Yet, the number of patients in the multivariate analysis was smaller (N = 1400), due to the fact that certain patients had missing covariates.

**Table 2:** Association of CCR5delta32 with different HCV-related endpoints.

	CCR5delta32^1^					
Variable	absent	%	present	%	OR (95% CI)	P
Spontaneous clearance^2^						
No	1073	0.83	217	0.17	Reference	
Yes	143	0.89	17	0.11	0.59 (0.35–0.99)	0.047
Sustained virological response						
No	228	0.83	47	0.17	Reference	
Yes	340	0.81	78	0.19	1.11 (0.75–1.66)	0.6
Fibrosis stage (Metavir)						
F0-F1	372	0.84	71	0.16	Reference	
F2-F4	472	0.82	104	0.18	1.15 (0.83–1.60)	0.4
Fibrosis progression rate						
<0.08	378	0.82	82	0.18	Reference	
≥0.08	388	0.84	74	0.16	0.88 (0.62–1.24)	0.5
Inflammation						
A0	359	0.86	61	0.15	Reference	
A1-A3	480	0.81	114	0.19	1.38 (0.99–1.95)	0.06
A0-A1	700	0.83	139	0.17	Reference	
A2-A3	137	0.79	37	0.21	1.36 (0.91–2.04)	0.1
Steatosis						
absent	252	0.83	52	0.17	Reference	
present	538	0.82	115	0.18	1.04 (0.72–1.49)	0.8

1 Due to the relative low CCR5delta32 allele frequency (8.6%), data are presented for the dominant mode of inheritance.

2 The number patients carrying 0, 1 or 2 copies of CCR5delta32 were 1073, 202 and 15 for patients with chronic infection and 143, 16 and 1 for spontaneous clearers. The associations were also tested by using the additive (OR = 0.61, 95% CI 0.38–1.00, P = 0.05) and recessive (OR = 0.53, 95% CI 0.07–4.07, P = 0.5) modes of inheritance. The association was similar albeit less significant when patients with positive HIV serology were removed (OR = 0.63, 95% CI 0.36–1.08, P = 0.09).

**Table 3:** Factors associated with spontaneous HCV clearance.

	Univariate		Multivariate	
	OR (95% CI)	P	OR (95% CI)	P
CCR5delta32 (dominant)	0.59 (0.35–0.99)	0.047	0.60 (0.35–1.04)	0.07
*IFNL3* rs12979860 (dominant)	0.36 (0.27–0.51)	5.9E-9	0.35 (0.24–0.49)	2.8E-09
Male gender	0.71 (0.51–0.99)	0.04	0.76 (0.53–1.07)	0.12
Risk factors				
Drug use	Reference		Reference	
Invasive procedure, needle stick	1.77 (1.10–2.86)	0.02	1.61 (0.98–2.63)	<0.001
Blood transfusion	3.04 (1.92–4.80)	<0.001	2.91 (1.81–4.66)	0.06
Other/missing	3.47 (2.14–5.61)	<0.001	3.55 (2.17–5.81)	<0.001

### Other phenotypes

Carriage of the CCR5delta32 allele was not associated with SVR (P = 0.6), fibrosis stage (P = 0.4), fibrosis progression rate (P = 0.5), or steatosis (P = 0.8). However, it tended to be observed more frequently among patients with liver inflammation (19%) compared to those without inflammation (15%, OR = 1.38, 95% CI interval 0.99–1.95, P = 0.06) (Table 2). However, the trend was weaker in a multivariate model (OR = 1.35, 95% CI 0.91–2.01, P = 0.14), after adjustment for *IFNL3 rs12979860* (P = 0.08), age at infection (P = 0.008) and steatosis (P<0.001) (Table 4).

**Table 4:** Factors associated with liver histological activity.

	Univariate		Multivariate	
	OR (95% CI)	P	OR (95% CI)	P
CCR5delta32 (dominant)	1.38 (0.99–1.95)	0.06	1.35 (0.91–2.01)	0.14
*IFNL3* rs12979860 (dominant)	0.76 (0.58–0.99)	0.04	0.76 (0.55–1.03)	0.08
Male gender	1.43 (1.11–1.85)	0.006		
Age at infection (continuous)	1.02 (1.01–1.04)	<0.001	1.02 (1.01–1.04)	0.008
Log HCV RNA IU/ml(continuous)	0.86 (0.76–0.98)	0.02		
Steatosis	2.33 (1.76–3.10)	<0.001	2.36 (1.73–3.22)	<0.001
HCV genotype				
1	Reference			
2	1.47 (0.93–2.33)	0.01		
3	1.32 (0.98–1.77)	0.07		
4	0.82 (0.53–1.25)	0.4		
Other/missing	1.03 (0.35–3.01)	1.0		

Note: stepwise logistic regression (cutoff P = 0.15).

## Discussion

Our study is based on the largest cohort of Caucasian anti-HCV positive patients with the most exhaustive determination of CCR5delta32 and other variables potentially associated with HCV clearance. With 15.1% of them being heterozygous and 1.1% homozygous for CCR5delta32, it is representative of the expected distribution of this allele in a Caucasian population.

The aim of our study was to clarify the potential role of CCR5delta32 in the outcome of HCV infection. With the new perspective of treatment of HCV-HIV co-infected patients with CCR5 antagonists such as maraviroc, a current anti-HIV therapy, this is an important question to debate in terms of safety and efficacy.

Firstly, we analysed the relationship between CCR5delta32 and HCV spontaneous clearance. By univariate analysis, HCV clearance was negatively associated with homo- or heterozygous CCR5delta32, polymorphisms at *IFNL3 rs12979860*, male gender, and positively associated with HCV acquisition through intravenous drug use, invasive procedures and especially by blood transfusion. Considering the possibility of some bias induced by co-infection with HIV and by the interaction between HIV and CCR5, we performed the same analysis after exclusion of co-infected patients: the association between CCR5 deletion and HCV spontaneous clearance was similar albeit less significant when patients with positive HIV serology were removed (P = 0.09). The association between the CCR5delta32 and HCV clearance barely lost statistical significance after adjustment for *IFNL3 rs12979860* and male gender. This observation matches the hypothesis that a reduced expression of CCR5 at the cell surface would impair the recruitment of Th1-expressing cells into the liver to mediate the clearance of HCV-infected hepatocytes, promoting persistence of HCV infection [15]–[17].

The currently available data on the frequency of CCR5delta32 in HCV-infected *vs* -cleared individuals are not unequivocal. Our results, based on a large cohort of 1,450 patients, are in conflict with some smaller studies. A recent Egyptian study enrolling 190 HCV-infected patients, mainly with HCV genotype 4 and coinfected with *Schistosoma mansoni*, showed a highly significant positive association between spontaneous HCV clearance and CCR5delta32 [22]. In another series of 283 female patients with HCV genotype 1, Goulding *et al.* showed an association between CCR5delta32 and an increased rate of spontaneous clearance [23]. In more recent work, on the other hand, Nattermann *et al*. [24] evaluated a cohort of 396 Caucasian female patients infected with a single source of HCV genotype 1 and showed that both *IFNL3 rs1297860* CT/TT and CCR5delta32 variants were independently associated with a decreased rate of spontaneous HCV clearance. An increased prevalence of CCR5delta32 homozygosity was reported in haemophilic patients with chronic HCV infection, with or without HIV coinfection, compared with the general population [25], suggesting that CCR5delta32 may favor HCV persistence. Thus, considering all these data together, if on the one hand patients with HCV infection and CCR5delta32 may be protected against HIV, on the other hand the functional blockade of CCR5 with drugs (e.g. maraviroc) in HIV-infected people may be associated with increased risk of progression to persistence in case of primary HCV infection.

Based on the assumption that a decreased expression of CCR5 impairs the Th1 response, we assessed whether the CCR5delta32 allele may influence the response to pegylated IFN-alpha and ribavirin therapy. The efficacy of IFN-alpha has been attributed to its ability to partially up-regulate CCR5 expression on T cells [26]. Our results show that this *CCR5* polymorphism did not play any significant role in the anti-HCV treatment response, while at the same time confirming the very well known role played by severe fibrosis, older age, higher pretreatment HCV RNA, genotype 2 and 3 and *IFNL3/4* genetic polymorphisms. As already known, the latter are currently the strongest host genetic markers to predict IFN-alpha-based treatment-induced HCV clearance, and, as demonstrated in a recent and large serie of 813 Caucasian patients with chronic hepatitis C genotype 1 [27], CCR5delta32 did not improve prediction of SVR in the context of the *IFNL3* polymorphisms. These results match those of previous, smaller series [28]–[31], but appear in conflict with those of an early study which had identified CCR5delta32 carriage as an independent negative predictor for the end of treatment virological response to the less potent IFN-alpha monotherapy [30]. This suggests that the role of CCR5delta32 may no longer be relevant in predicting the treatment-induced clearance of HCV when more powerful regimens are used.

If CCR5 is partly responsible for the recruitment of Th1 cells, a decreased expression of CCR5 should result in milder inflammation, as showed by Hellier and Goulding [18], [22].

Immunohistochemical analysis of liver biopsies of patients chronically infected with HCV genotype 1 showed a significant positive correlation between the percentage of intrahepatic CCR5 high-expressing CD8+ cells and both porto-periportal and lobular activity [32].

Other studies [31], [33] reported a lack of correlation between CCR5 deletion and liver inflammation. Our data show a trend towards increased inflammation in the presence of CCR5delta32. Considering the relationship between CCR5 and TH1 cells, this observation is difficult to explain. However, it is known that CCR5 is not the only chemokine receptor involved in Th1 response [32], [34]: also CXCR3 and CXCR6 play a major role in recruiting cytotoxic T cells and secreting type 1 cytokines in the liver, and it is likely that, in case of the *CCR5* deletion, other chemokine receptors may contribute to the inflammatory response. The positive correlation between CCR5delta32 and inflammation was found only when considering A0 vs A1-3 but disappeared when considering A0-1 vs A2-3, suggesting that *CCR5* deletion probably does not play any role in significant inflammation.

Finally, we aimed to study fibrosis and fibrosis progression rate as a function of CCR5 expression. CCR5 is recognised as an important mediator of pro-fibrogenic signalling in HSCs and the *CCR5* deletion should protect against fibrosis progression. Recently, Bruno *et al.* [14] demonstrated that the HIV envelope glycoprotein gp120 directly acts on HSCs through the use of the CCR5 receptor. The link between homozygous CCR5delta32 and HCV fibrogenesis has been analysed in several studies. *CCR5*-deficient mice displayed reduced hepatic fibrosis and macrophage infiltration [35]. Hellier et al [18] evaluated a cohort of 623 HCV-infected patients and found a significant association between the CCR5delta32 homozygous genotype and reduced portal inflammation and milder fibrosis.

Most of the other studies failed to demonstrate any significant relationship between the CCR5delta32 mutation and the course of chronic hepatitis C [22], [28], [31], [33]. Our work did not identify any association between CCR5delta32 and liver grading or staging. When considering only CCR5delta32 homozygous patients, severe and significant fibrosis were found in 8 and 25% of patients, respectively, *vs* 28 and 54% in patients with the wild type *CCR5*. However the number of homozygous patients was too small to reach any statistical significance. As already suggested by several studies [33], [36]–[39], the use of CCR5 antagonist such as maraviroc, should be safe in patients with HIV and chronic hepatitis C, at least as far as liver histology and the efficacy of anti-HCV treatment are concerned. A pilot clinical trial suggested that maraviroc may even reduce the liver fibrosis progression in HIV-HCV coinfected patients [39]. Our data do not support a potential antifibrotic effect of CCR5delta32, perhaps due to the small number of CCR5delta32 homozygous patients and also to the prevailing effect of other pro-fibrogenic variables. Only a large randomized prospective study might assess this effect of the CCR5 antagonist, if any. An anti-HIV investigational drug, the cenicriviroc, being both a CCR5 and CCR2 inhibitor, demonstrated good antiviral activity and tolerability in a Phase 2 clinical trial. Whether this new anti-inflammatory activity on the top of a CCR5 blockage could be efficient to decrease fibrosis rate in HCV- (co-) infected patients remains to be proven.

In conclusion, our results, based on a large cohort of 1,450 HCV-infected patients, genotyped for the CCR5delta32 allele and representative of the distribution of this allele in a Caucasian population, showed that that both *IFNL3 rs1297860* CT/TT and CCR5delta32 alleles were associated with a decreased spontaneous HCV clearance, although the multivariate analysis barely failed to reach statistical significance for the latter. The CCR5delta32 deletion was not associated with fibrosis, fibrosis progression rate, or therapy response. In the view that CCR5 inhibitors are now available for HIV treatment, this is an important observation: our data suggest that these drugs could impair the odds of spontaneous clearance of acute HCV infection in HIV-infected patients on active treatment with anti-CCR5. However drug-induced impairment of CCR5 signaling should neither modify HCV histological outcomes nor impair the efficacy of anti-HCV therapy.
